# PB1F2 from Influenza A Virus Regulates the Interaction between Cytochrome C and Cardiolipin

**DOI:** 10.3390/membranes12080795

**Published:** 2022-08-18

**Authors:** Yujuan Wang, Junfeng Wang

**Affiliations:** 1 High Magnetic Field Laboratory, CAS Key Laboratory of High Magnetic Field and Ion Beam Physical Biology, Hefei Institutes of Physical Science, Chinese Academy of Sciences, Hefei 230031, China; 2 Institutes of Physical Science and Information Technology, Anhui University, Hefei 230601, China

**Keywords:** PB1F2, cytochrome c, cardiolipin, liposome, nanodisc

## Abstract

PB1F2 is a membrane associated protein encoded by the influenza virus gene in the host. Similar to endogenous pro-apoptotic proteins, it acts on the mitochondria of the host immune cells, inducing apoptosis of the cells. The PB1F2 protein has been demonstrated to facilitate the release of cytochrome c in addition to impairing the integrity of the inner mitochondrial membrane. This investigation focused on how the protein PB1F2 interacted with cardiolipin and cytochrome c. The regulation of PB1F2 on the binding of cytochrome c to cardiolipin in two kinds of in vitro membrane mimics was investigated by biophysical techniques. PB1F2 aids in the dissociation of cytochrome c-cardiolipin complexes in liposomes and nanodiscs. The results provide novel explanations and evidence for how PB1F2 functions as a viral virulence factor by inducing immune cell death.

## 1. Introduction

Apoptosis begins when cells are stressed by outside causes like viruses in addition to the appropriate immunological response [1,2,3,4]. PB1F2 is a membrane protein encoded by influenza virus genes and expressed in the host. Since its discovery in 2001, PB1F2′s localization and function within the host cell have been continuously revealed [5,6,7,8]. Additionally, the molecular functions of the PB1F2 protein include inhibiting the natural immune response against the virus, promoting the production of pro-inflammatory cytokines, enhancing viral polymerase activity, and inducing apoptosis [9]. PB1F2, which is localized to the mitochondria of the host’s native immune cells, eventually induces their apoptosis, disrupting the host’s immune system delaying the clearance of the virus [6,10,11,12]. Synthetic PB1F2 appears typical of a largely random coil peptide in aqueous solutions but tends to fold to α-helical secondary structure when liposomes are added [13,14]. PB1F2 proteins expressed in inclusion form can refold in a solution of pH5, but the refolded soluble PB1F2 proteins still behave as structureless. The addition of membrane systems can cause the formation of fibrous aggregation of PB1F2 proteins, which is thought to play a significant role in the destruction of mitochondrial membranes by PB1F2 [15,16,17,18,19].

PB1F2 enters intermembrane space between the inner and outer membranes through the TOM40 transport channel of the outer mitochondrial membrane. Studies have shown that PB1F2 aggregates or forms fibrillar aggregates on the mitochondrial membrane, and eventually destroys the inner mitochondrial membrane [20,21,22]. However, PB1F2 leads to increased cell sensitivity to apoptosis stimuli by interacting with the apoptosis regulator of the mitochondrial adenine nucleotide translocator 3 (ANT3) and the voltage-dependent anion channel l (VDACI) outside the mitochondrial membrane. PB1F2 may exert its pro-apoptosis effect through mitochondrial permeability transition pore complex (PTPC). During this process, cytochrome c is the key molecule that mediates PB1F2 and apoptosis. Additionally, the release of cytochrome c by addition of the PB1F2 protein to cultured cells or extracted mitochondria also suggests that the apoptosis process induced by the viral protein PB1F2 is initiated by cytochrome c [5,11].

Cytochrome c is a water-soluble protein containing heme, located between the inner and outer membranes of mitochondria. Cytochrome c plays an important physiological function in mitochondrial respiration and apoptosis [23,24,25]. When cells are exposed to endogenous stressors such as DNA damage and the activation of cancer genes, a portion of cytochrome c is finally released into the cytoplasm. [26,27].

Except for the portion of cytochrome c involved in the transmission of the respiratory chain, the majority of cytochrome c in cells is bound to the inner mitochondrial membrane by cardiolipin under normal physiological conditions. The complicated interactions between cytochrome c and the phospholipid membrane include hydrophobic interactions, electrostatic interactions, and changes in membrane curvature associated with phospholipid composition and conformational changes in the protein itself. Cardiolipin is a characteristic phospholipid molecule in the mitochondrial inner membrane. Cardiolipin binds to multiple positions of cytochrome c, including A site, CXXCH motif, etc. The binding of cytochrome c to cardiolipin induces its conformational switch to unfold, which facilitates cardiolipin insertion into cytochrome c for peroxidation. As soon as cardiolipin is oxidized, the interaction between the two weakens and cytochrome c is released [23,28,29,30,31,32,33,34].

According to the results of PB1F2 and the interaction between cytochrome c and cardiolipins, we speculate that the PB1F2 protein encoded by the influenza virus gene enters intermembrane space through the TOM40 of the outer membrane, assisting/promoting the dissociation of cytochrome c and cardiolipin binding on the inner membrane. Then, the released cytochrome c enters the cytoplasm and induces apoptosis of immune cells. Based on this, we use two kinds of membrane mimic system containing cardiolipin, liposome and nanodisc, to study the regulation of PB1F2 on the interaction between cytochrome c and cardiolipin. The results provide some evidence for explaining the molecular mechanisms by which PB1F2 induces apoptosis in immune cells from the mitochondria level as a toxicity factor.

## 2. Materials and Methods

### 2.1. Bacterial Strains, Plasmids, Media, and Chemicals

*E. coli* strain BL21 (DE3) was used as the host for protein expression. The lipids POPC (1-palmitoyl-2-oleoyl-sn-glycero-3-phosphocholine), NBD-PE (1-oleoyl-2-{12-[(7-nitro-2-1,3-benzoxadiazol-4-yl) amino] dodecanoyl}-sn-glycero-3-phosphoethanolamine), cardiolipin, and CHAPS were purchased from Avanti Polar Lipids (Alabaster, AL, USA). Horse cytochrome c was purchased from Sigma (Merck KGaA, Darmstadt, Germany). Isopropyl-β-D-thiogalactopyranoside (IPTG) and all the other chemicals were from Sangon Biotech (Shanghai, China).

#### Protein Expression and Purification

PB1F2 and deletion mutation of membrane scaffold protein (ΔMSP) were all expressed in BL21(DE3) cells under the control of the T7 promoter (pET28a and pET22a), purified as described previously [22,35]. Purified protein was stored in 20 mM PB (K2HPO4/KH2PO4) 100 mM NaCl, pH7.2 buffer in an −80 °C refrigerator.

### 2.2. Preparation of Nanodiscs

The general steps for self-assembling nanodiscs are the same as those previously described [35]. In short, the optimal molar ratio, previously calculated from pre-experiments, was obtained by combining the solution of pure ΔMSP at 0.15–0.3mM concentration with cholate-solubilized phospholipid (POPC or POPC+cardiolipin). [36,37]. For every 1 mL of the MSP and phospholipid-sodium cholate mixture, about 0.6 g of bio-beads were added. The incubation temperatures were set according to the phase transition temperature of the phospholipid components (The incubation temperatures for POPC nanodisc and POPC+cardiolipin nanodisc are 23 °C and 28 °C). Additionally, the incubation period lasted at least 4 h. Finally, a Superdex 200 column with gel filtration was used to clean the nanodiscs (GE Healthcare, Uppsala, Sweden).

The assembly of nanodiscs containing NBD-PE was similar to that described above. The difference is that 0.1% (mass ratio) NBD-PE is added to the phospholipid (PC+CL) dissolved in cholate. Protect the lipids from exposure to ambient light by aluminum foil in order to minimize the photobleaching of dyes.

### 2.3. Liposome Pull down Assay

Liposome pulldown assay was carried out as described in the literature [38]. The major scaffold lipid chosen to replicate a membrane bilayer was POPC. The combined phospholipids with the required compositions were dissolved in chloroform in a round-bottom flask to prepare the liposomes. To create a thin layer, the solvent was evaporated under a stream of nitrogen gas. The dried lipid mixture was then further vacuum-pumped lyophilized for an additional 16 h, hydrated with extrusion buffer (20 mM potassium phosphate buffer, 100 mM NaCl, pH 7.2), then repeatedly frozen and thawed in liquid nitrogen and a 37 °C water bath. The hydrated lipid mixture was extruded through a 0.1 um polycarbonate filter (Avanti Polar Lipids Inc., Alabaster, AL, USA) to create liposomes.

The tested protein was ultracentrifuged at 70,000 rpm (218,400× *g*) for 30 min to eliminate pellets before the binding tests. For the binding tests, a total volume of 100 μL of the indicated liposomes (80 μL) and the proteins (20 μL) were incubated at room temperature for 30 min. Following that, samples were centrifuged at 4 °C for 30 min at 70,000 rpm. The pellets were resuspended in 100 μL of extrusion buffer after the supernatant was transferred to a different tube. Following Coomassie blue staining, SDS-PAGE was used to investigate these two fractions. Analysis was performed with ImageJ software (National Institute of Health, Bethesda, MD, USA). The band density was quantified and expressed as the relative gray value.

### 2.4. Nanodisc-Based Fluorescence Detection

Fluorescence detection is accomplished by the fluorescent phospholipid NBD-PE (excitation/emission 460/535 nm) assembled in a nanodisc. As shown in Table 1, nanodisc control samples, mixed samples of nanodisc and cytochrome c and ternary mixed samples of nanodisc and cytochrome C and PB1F2 were prepared, respectively. All the samples underwent a second incubation with the addition of PB1F2 (or buffer) after being treated with cytochrome C for 30 min at room temperature. The fluorescence of the samples was read using a microplate reader (MOLECULAR DVICES, SpectraMax i3x, San Jose, CA, USA) after the second 30 min of incubation. The entire operation is protected from light.

### 2.5. SEC (Size Exclusion Chromatography) Analysis

Nanodisc control samples, mixed samples of nanodisc and cytochrome C, and ternary mixed samples of nanodisc and cytochrome C and PB1F2 were prepared as described in Section 2.4. All the samples underwent a second incubation (2 h) with the addition of PB1F2 (or buffer) after being treated with cytochrome C for 30 min at room temperature. All the SEC analyses were performed in FPLC (AKTApurifierTM UPC 10, Uppsala, Sweden) using Superdex 75 column (GE Healthcare, Uppsala, Sweden). The buffer used is 20 mM potassium phosphate buffer, 100 mM NaCl, pH 7.2.

### 2.6. Binding Affinity Measurements by Using Monolayer Technology

Utilizing a Microtrough from Kibron (Helsinki, Finland), the binding affinity between PB1F2/Cytochrome and cardiolipin was measured. The general measurement process is the same as what was previously detailed in the literature [39,40]. Briefly, until the initial surface pressure (∏i) was obtained, a few microliters of the phospholipid (PC+cardiolipin, the concentration of cardiolipin was the same as that of the liposome component) chloroform solution were dispersed at the surface. Protein was then introduced beneath the phospholipid monolayer to measure the rise in surface pressure. Protein adsorption onto phospholipid monolayers was monitored until equilibrium surface pressure (∏e) was reached. Using the data gathered, maximum insertion pressure (MIP) was determined. The MIP corresponds to the maximum surface pressure of the monolayer up to which the peptide can insert in the monolayer and beyond which no insertion takes place. The MIP of the proteins was determined by extrapolating the plot of the surface pressure increase (Δ∏) as a function of the initial surface pressure where the curve reaches a value of 0 on the *x*-axis. The surface pressure increase corresponds to Δ∏ = ∏e − ∏i.

### 2.7. Statistics and Reproducibility

All experiments were independently repeated at least three times. The statistical significance of differences was determined by using Student’s *t*-test with GraphPadPrism 8 (GraphPad Software, San Diego, CA, USA) for comparison between two groups, and ANOVA for comparison among multiple groups. Probability (*p*) values < 0.05 were considered to be statistically significant differences. * *p* < 0.05, ** *p* < 0.01, *** *p* < 0.001. NS is not significant.

## 3. Results

### 3.1. Interaction of PB1F2 and CytC with Cardiolipins

First of all, we use liposomes to study the interaction of PB1F2 and cytochrome c proteins with phospholipid membranes. We assembled liposomes containing (or without) cardiolipins according to the component content of membrane phospholipids in the mitochondria. The liposome size is about 100 nanometers, and the content of cardiolipin is 20% (mass ratio). Liposome pulldown was used to assay the interaction between cytochrome c and PB1F2 proteins and cardiolipins. After mixing and incubating different kinds of liposomes with proteins, membrane-bound proteins are separated by ultracentrifugation from the unbound. The BCA assay and SDS-PAGE were used to detect the amount of the binding of the two proteins to liposomes.

**Figure 1:**
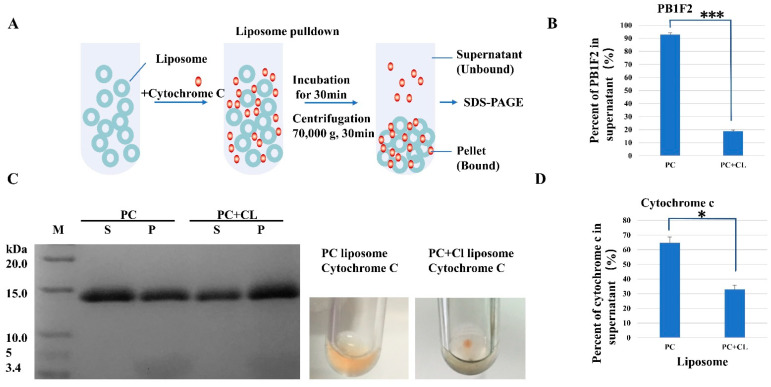
Characterization of the phospholipids binding specificity of Cytochrome C. (**A**) schematic diagram of the liposome pulldown assay; (**B**) BCA assay results of lipsosome pull down of PB1F2; (**C**,**D**) SDS-PAGE and quantification of liposome pulldown results of Cytochrome C. Liposomes were incorporated with POPC (PC) or POPC and cardiolipin (PC+Cl). Liposomes (1 mg/mL) and cytochrome c (0.2 mg/mL) were incubated at room temperature for 30 min. After ultracentrifugation, the pellet (P) and supernatant (S) were analyzed by SDS-PAGE. Data were pooled from three independent experiments, and the results were represented as mean ± SD. * *p* < 0.05, *** *p* < 0.001.

If cytochrome c or PB1F2 binds to liposomes, it will be pulled down with liposomes to the bottom of the test tube. In addition, the proteins remaining in the supernatant are proteins that are not bound to liposomes. The results of the BCA assay showed that a minority of PB1F2 bind to liposomes containing only PC, and the PB1F2 content in the supernatant was 91.8 ± 1.27%. In contrast, the majority of PB1F2 bind to liposomes containing PC and cardiolipin (Figure 1B). The content of PB1F2 in the supernatant reduced to 18.9 ± 0.95% indicates the interaction between cardiolipins and PB1F2. The interaction may result from the electrostatic interactions between positive charged PB1F2 and anionic phospholipid cardiolipin.

As far as cytochrome c is mentioned, the SDS-PAGE picture (Figure 1C,D) shows the similar phenomenon. Compared with the interactions with PC only liposomes, the reduction of cytochrome c in the supernatant illustrates that more cytochrome c bind to the liposomes containing PC and cardiolipin. This is consistent with the results previously reported [32]. The interaction between SNX16 (Sorting nexins 16, domain of SNX16 specifically binds to phosphatidylinositol 3-monophosphate) and PC liposome was used as negative control. There is almost no interaction between PC liposome and SNX16 (Figure S1 in the Supporting Information).

### 3.2. PB1F2 Promotes the Dissociation of Cytochrome c from Cardiolipins

#### 3.2.1. Liposome Pulldown

**Figure 2:**
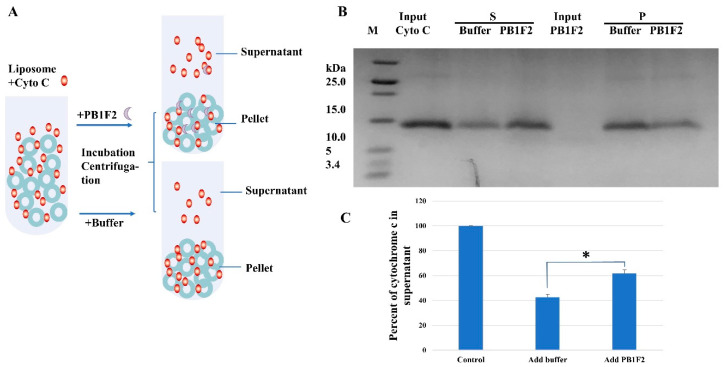
PB1F2 regulates the interaction between cytochrome c (Cyto C) and cardiolipin in liposome. (**A**) schematic diagram of the liposome pulldown assay; (**B**,**C**) SDS-PAGE and quantification of the liposome pull down assay with PB1F2. PB1F2 assist with the dissociation of cytochrome C with cardiolipin. Liposomes (1 mg/mL) were incorporated with POPC or POPC, and cardiolipin. PB1F2 (0.2 mg/mL) was added to the cytochrome C and liposome mixture after 30 minutes of incubation at room temperature. After ultracentrifugation, the pellet (P) and supernatant (S) were analyzed by SDS-PAGE. ‘Input cyto C’ and ‘Input PB1F2’ refer to cytochrome c protein and PB protein added to liposome. Data were pooled from three independent experiments, and the results were represented as mean ± SD. * *p* < 0.05.

On the basis of the interaction between cardiolipin and cytochrome c, we introduced the PB1F2 protein to test whether PB1F2 can induce the dissociation of cytochrome c and cardiolipin. During the liposome pulldown, a certain amount of PB1F2 was added to the liposome–cytochrome c mixture after half an hour of incubation. Ultracentrifugation was carried out after another 30 minutes of incubation. As can be seen from Figure 2, PB1F2 was able to dissociate cytochrome c bound to cardiolipin into solution. When PB1F2 protein was added to the complex system of cytochrome c and liposome with cardiolipin, compared with the treatment with the addition of buffer, the cytochrome c in the supernatant increased and the cytochrome c in the pellet (fraction bound to the liposome) decreased.

It can be speculated that, after PB1F2 is expressed in host cells, most of it enters the space between the inner and outer membranes of the mitochondria. A part of PB1F2 destroys the inner mitochondrial membrane by interacting with negatively charged phospholipids in the inner mitochondrial membrane, and a part of PB1F2 functions through fibrillar aggregation in the inner mitochondrial membrane. At the same time, PB1F2 could also cause cytochrome c and cardiolipin to separate. We hypothesize that PB1F2 may increase the apoptosis level of immune cells by promoting the dissociation of cytochrome c. The PB1F2 protein has numerous roles via different states and may engage in many functional modes simultaneously or at various stages of virus infection [16,22].

#### 3.2.2. Nanodiscs Assay

**Figure 3:**
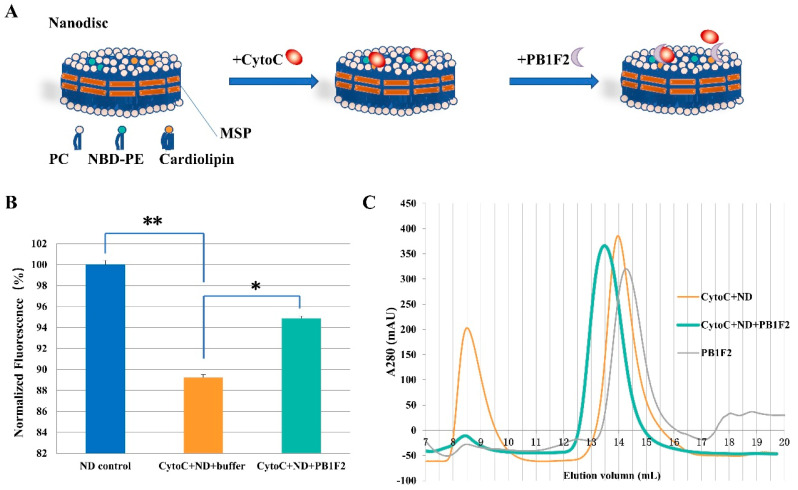
PB1F2 regulate the interaction between cytochrome c (Cyto C) and cardiolipin on Nanodiscs (ND). (**A**) schematic diagram of the nanodiscs fluorescence assay; (**B**) interaction of cytochrome c and PB1F2 with cardiolipin leads to the difference in nanodisc fluorescence. Nanodiscs were incorporated with POPC, cardiolipin, and NBD-PE. PB1F2 was added to the cytochrome C and liposome mixture after 30 minutes of incubation at room temperature. The fluorescence of the samples was recorded at excitation/emission 460/535 nm using a microplate reader. Data were pooled from three independent experiments and the results were represented as mean ± SD. * *p* < 0.05, ** *p* < 0.01. (**C**) The adding of PB1F2 to the mixture of cytochrome C and nanodisc (ND) resulted in a larger molecular weight component (Green). SEC analysis was carried out after 30 minutes of incubation.

The results listed above mainly used liposomes to study the regulation of PB1F2 protein on the binding of cytochrome c on cardiolipin. We also validated those results using phospholipid bilayer nanodiscs assembled with ΔMSP. Nanodiscs are relatively monodisperse model membranes with lipid bilayer comprised of synthetic lipids. The use of ΔMSP stabilizes the edge of the mimic membranes. Compared with liposme, nanodiscs are more homogeneous in size and more stable membrane mimic [35]. Besides the specific cardiolipin in the mitochondrial inner membrane, PC and PE are the two most important phospholipid components. According to the composition ratio of phospholipid components in the inner membrane of mitochondria, we add fluorescently labeled phospholipids (NBD-PE) to the phospholipids (PC+Cardiolipin) for nanodisc assembling (Figure 3A).

Similar to the experimental procedure of liposome pulldown above, the fluorescently labeled nanodisc was first incubated with cytochrome c, followed by the addition of PB1F2 protein or the appropriate buffer, and finally the interaction between the protein and the membrane system was detected by fluorescence. As can be seen from Figure 3B, the inclusion of cytochrome c decreased the fluorescence value of the system in comparison to the nanodisc control system, once more illuminating the relationship between cardiolipin and cytochrome c. Since NBD-PE and cardiolipin are uniformly distributed in nanodisc, the binding of cytochrome C to cardiolipin affects the fluorescence of nearby NBD-PE [41,42]. Compared with mixed systems including cardiolipin nanodisc and cytochrome c, the presence of PB1F2 protein increased the fluorescence in the system. The increase indicates that PB1F2 promotes the dissociation of cytochrome c linked to cardiolipin.

The fluorescence increase by PB1F2 illustrates that the fluorophore that had been covered by cytochrome c coupled to cardiolipin had been released. The interaction between PB1F2 and cytochrome c may be the combination of PB1F2 and cytochrome c to form a novel complex. Cytochrome c’s interaction with cardiolipin is altered by the complex, which is followed by cardiolipin dissociation. Another possibility is that the binding of PB1F2 to cardiolipin is stronger than that of cytochrome c, which competes with cytochrome c bound to cardiolipin.

### 3.3. SEC Analysis

According to the regulation of PB1F2 protein on the interaction between cytochrome c and cardiolipin obtained using liposome and nanodisc, it can be inferred that PB1F2 can promote the dissociation of cytochrome c and cardiolipin. By using SEC, we also investigated the interaction of cytochrome c, PB1F2, and nanodisc containing cardiolipin. As shown in Figure 3C, PB1F2 was eluted at approximately 14.2. Sample of cytochrome c and nanodisc shows two peaks at 14 mL and 8.5 ml, corresponding to free cytochrome c and nanodisc-bound cytochrome c. An obvious protein peak appeared near 13.5 mL after the PB1F2 protein was added to the mixed system of Nanodisc and cytochrome c. The molecular weight is slightly larger than that of PB1F2 or cytochrome c monomer. The component corresponding to the larger molecular weight may be a heterodimer of PB1F2 and cytochrome.

### 3.4. LB Microtrough Analysis

**Figure 4:**
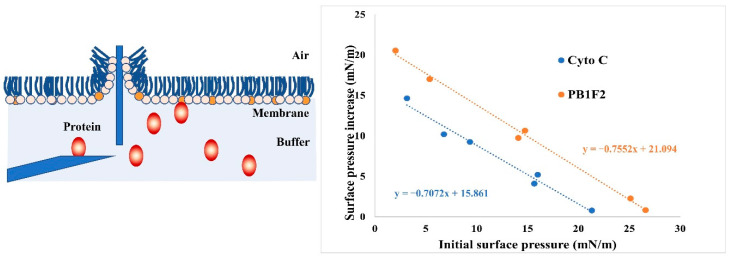
Schematic diagram and results of LB Microtrough analysis. The PB1F2 or cytochrome c was injected underneath the monolayer (PC+cardiolipin). MIPs of PB1F2 and cytochrome c (Cyto C) are calculated from lines fitted in this graph.

Since PB1F2 is able to regulate the binding of cytochrome c to cardiolipin in the membrane, we hypothesize that a cause is the different affinities of PB1F2, cytochrome c, and cardiolipin. Here we use the Langmuir–Blodgett Trough (Micro Trough X, Kibron, Finland) to detect the binding affinity of the two protiens to cardiolipins. The determination of the MIP can provide information on the affinity of a peptide for a given type of lipid as well as on its extent of binding to membranes [40]. PC and cardiolipin were chosen as the phospholipid components of the phospholipid monolayers. The PB1F2 protein and cytochrome c protein were injected underneath the monolayer respectively, and the data were gathered simultaneously. Use initial surface pressure and surface pressure increase to plot and fit the curve in the figure to calculate MIP (mN/m). According to Figure 4, the MIP of cytochrome c was 22.43 ± 1.9 mN/m and the MIP of PB1F2 was 27.93 ± 2.2 mN/m, demonstrating that PB1F2 had a greater affinity for cardiolipin than did cytochrome c.

## 4. Discussion

PB1F2 is a toxic protein encoded by viral genes after the virus infects the host. Studies have shown that PB1F2 may enhance the toxicity of the virus by promoting apoptosis of immune cells through mitochondrial pathway [43].

Numerous investigations have been done on PB1F2 and mitochondria. It has been described that PB1-F2 is able to inhibit the host response, especially through its inhibitory action on MAVS [12,44]. Another study revealed that PB1-F2 induced complete mitophagy [45]. Likewise, the results of investigations on PB1F2 and cytochrome c are also important findings. Experimental studies at the cellular level have shown that adding PB1F2 protein to cells can promote the release of cytochrome c [5]. The mitochondrial cytochrome c was also released when the isolated mitochondria were treated with PB1F2 for a while [11]. These outcomes are consistent with what we discovered in vitro. We characterized the interaction of PB1F2 and cytochrome c with cardiolipin in vitro using a simulated membrane system containing cardiolipin. Early in vitro studies targeting PB1F2 mainly utilized solid-phase synthesis. The synthesized protein has the function of disrupting the liposome (PC + PE) [46]. Some researchers also used refolded PB1F2 for the study of conformational changes in the environment of anionic liposomes [15]. In contrast to the phospholipid components previously used, a membrane system incorporating cardiolipins was used in the study of PB1F2.

The results of the microtrough studies and the liposome pull down assay indicated that the binding of PB1F2 and cytochrome C to cardiolipin was different. It is necessary to use more reliable experimental techniques to demonstrate the affinity of PB1F2 and cytochrome C to cardiolipin. The liposome pull down and fluorescence experiments with PB1F2 indicated that PB1F2 promoted the dissociation of cytochrome C and cardiolipin. The assumption needs to be clarified by further investigations that focus on the interaction interface and key amino acids between PB1F2 and cytochrome c.

On one hand, PB1F2 may stimulate cytochrome c release while competing with cardiolipin for the binding of cytochrome c. On the other hand, PB1F2 destroys the integrity of the inner mitochondrial membrane by interacting with anionic phospholipids and promotes apoptosis [22]. When the number of subsequent viruses increases, a large amount of PB1F2 protein accumulates in the mitochondrial intermembrane space, which may lead to fibrillar aggregation of PB1F2 protein and consequent mitochondrial damage [15]. Of course, these processes also include a series of responses at the level of immunity induced by PB1F2 [12,44,47]. The detailed mechanism underlined that it needs to be further studied by infecting cells/mice with viruses containing the PB1F2 gene.

## Figures and Tables

**Table 1:** Components of the mixture for nanodisc-based fluorescence detection.

Sample	Nanodisc	Cytochrome c	PB1F2	Buffer
ND	+	-	-	++ ^1^
ND+Cyto C	+	+	-	+ ^2^
ND+Cyto C+PB1F2	+	+	+	-

^1^ refers to same volume of cytochrome c buffer and PB1F2 buffer. ^2^ refers to same volume of PB1F2 buffer. + and - represent the present or absence of the component, respectively.

## Data Availability

The data presented in this study are available in the main text and the Supplementary Materials.

## Supplementary Materials

The following supporting information can be downloaded at: https://www.mdpi.com/article/10.3390/membranes12080795/s1. Figure S1: SDS-PAGE of liposome pulldown results of SNX16 and PC liposome. Figure S2: Dynamic Light Scattering (DLS) analysis of nanodiscs.
