# Supplementary material for: PB1F2 from Influenza A Virus Regulates the Interaction between Cytochrome C and Cardiolipin
Source: Membranes (Basel). 2022 Aug 18;12(8):795. doi: 10.3390/membranes12080795 (PMC9414537; doi:10.3390/membranes12080795)
Supplement: Supplementary file 1 [file membranes-12-00795-s001.zip › membranes-1833999-supplementary.pdf]

membranes

MDPI

Supplementary Materials

PB1F2 from Influenza A Virus Regulate the Interaction between Cytochrome C and Cardiolipin

Yujuan Wang [1,*] and Junfeng Wang [1,2,*]

[1] High Magnetic Field Laboratory, CAS Key Laboratory of High Magnetic Field and Ion Beam Physical Biology, Hefei Institutes of Physical Science, Chinese Academy of Science, Hefei, Anhui 230031, China
[2] Institutes of Physical Science and Information Technology, Anhui University, Hefei, Anhui 230601, China
* Correspondence: yjwang@hmfl.ac.cn (Y.J.W.); Junfeng@hmfl.ac.cn (J.F.W.)

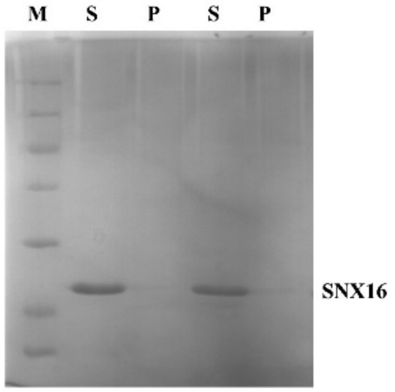

Figure S1. SDS-PAGE of liposome pulldown results of SNX16 and PC liposome. Liposomes and SNX16 were incubated at room temperature for 30 min. After ultracentrifugation, the pellet (P) and supernatant (S) were analyzed by SDS-PAGE.

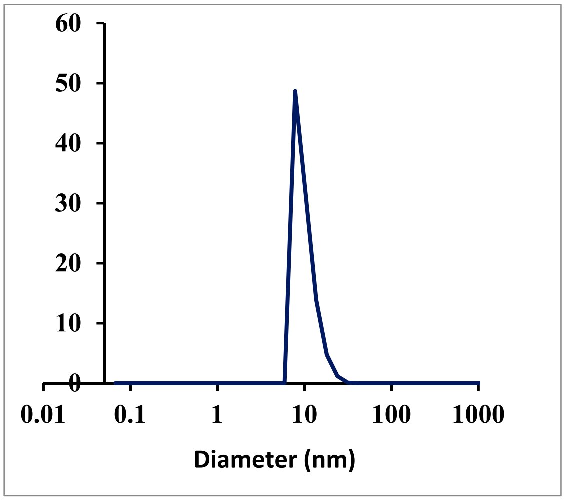

Figure S2. Dynamic Light Scattering (DLS) analysis of nanodiscs. Size measurements by DLS were carried out at 20 °C with DNYAPRO-99 (Wyatt). Fresh nanodisc samples from size exclusion chromatography fractions were used. DLS provides information about the sizes of nanodisc. Shown in Figure S2 is the DLS results from the nanodisc fractions after size exclusion chromatography. The diameter of nanodisc is about 10 nm.

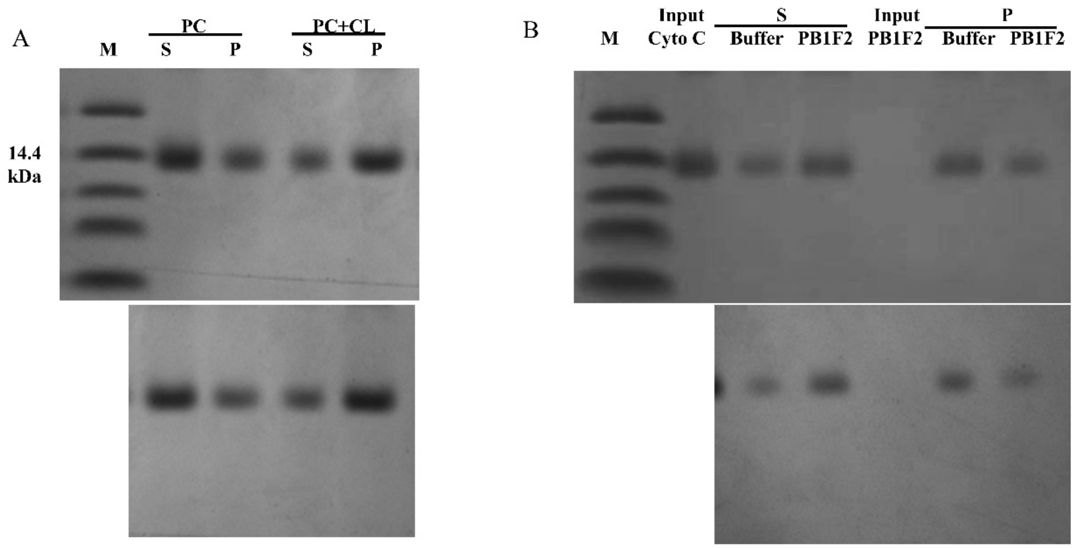

Figure S3. The replicate gels of liposome pull down assay. (**A**) SDS-PAGE of liposome pulldown results of Cytochrome C and PC/PC+cardiolipin liposome. (**B**) SDS-PAGE of the liposome pull down assay with PB1F2.
